# Selinexor inhibits growth of patient derived chordomas *in vivo* as a single agent and in combination with abemaciclib through diverse mechanisms

**DOI:** 10.3389/fonc.2022.808021

**Published:** 2022-08-18

**Authors:** Christopher J. Walker, Hua Chang, Leah Henegar, Trinayan Kashyap, Sharon Shacham, Josh Sommer, Michael J. Wick, Joan Levy, Yosef Landesman

**Affiliations:** ^1^ Department of Translational Research, Karyopharm Therapeutics, Inc, Newton, MA, United States; ^2^ Department of Research, Chordoma Foundation, Durham, NC, United States; ^3^ Department of Research, XenoSTART, San Antonio, TX, United States

**Keywords:** chordoma, CDK inhibition, proteasome inhibition, SINE inhibition, selinexor

## Abstract

Chordoma is a rare cancer that grows in the base of the skull and along the mobile spine from remnants of embryonic notochord tissue. The cornerstone of current treatments is surgical excision with adjuvant radiation therapy, although complete surgical removal is not always possible. Chordomas have high rates of metastasis and recurrence, with no approved targeted agents. Selinexor and eltanexor are selective inhibitors of nuclear export (SINE) that prevent the karyopherin protein exportin-1 (XPO1) from shuttling its cargo proteins through nuclear pore complexes out of the nucleus and into the cytoplasm. As cancer cells overexpress XPO1, and many of its cargos include tumor suppressor proteins and complexes bound to oncogene mRNAs, XPO1 inhibition can suppress oncogene translation and restore tumor suppressor protein activity in different cancer types. SINE compounds have exhibited anti-cancer activity in a wide range of hematological and solid tumor malignancies. Here we demonstrate the preclinical effectiveness of SINE compounds used as single agents or in combination with either the proteasome inhibitor, bortezomib, or the CDK4/6 inhibitor, abemaciclib, against various patient- derived xenograft (PDX) mouse models of chordoma, which included clival and sacral chordomas from adult or pediatric patients with either primary or metastatic disease, with either differentiated or poorly differentiated subtypes. SINE treatment significantly impaired tumor growth in all five tested chordoma models, with the selinexor and abemaciclib combination showing the strongest activity (tumor growth inhibition of 78-92%). Immunohistochemistry analysis of excised tumors revealed that selinexor treatment resulted in marked induction of apoptosis and reduced cell proliferation, as well as nuclear accumulation of SMAD4, and reduction of Brachyury and YAP1. RNA sequencing showed selinexor treatment resulted in differences in activated and repressed signaling pathways between the PDX models, including changes in WNT signaling, E2F pathways and glucocorticoid receptor signaling. This is consistent with SINE-compound mediated XPO1 inhibition exhibiting anti-cancer activity through a broad range of different mechanisms in different molecular chordoma subsets. Our findings validate the need for further investigation into selinexor as a targeted therapeutic for chordoma, especially in combination with abemaciclib.

## Introduction

In humans, the notochord is a transient structure present in embryonic development that is a major regulator of spatial patterning. The notochord releases critical signaling molecules such as sonic hedgehog (SHH) (1) to direct surrounding cells into structures including the neural tube. Later in life, primitive notochord cells, which have lodged within bones of the skull and spine can become oncogenic (2, 3). This results in a rare type of sarcoma called chordoma, which is clinically identified by overexpression of the T-box transcription factor, Brachyury (4). Chordomas can arise along the entirety of the spinal axis, with sacral accounting for about 50% followed by base of skull (30%) and spine (20%) (5, 6). Although pediatric cases exist, chordomas are predominantly diagnosed in older populations (7, 8). Standard frontline treatment includes aggressive surgical intervention (9, 10), which has a high mortality rate due to tumor integration within complex spinal and neurovascular architecture (9, 11). Adjuvant systemic chemotherapies and radiotherapy are frequently used (12–14). Despite recent advances, there remains a high rate of recurrence and metastasis unsuitable for further surgical intervention (9, 14, 15), and, therefore, alternative treatment options are urgently needed. Molecularly, chordoma growth is perpetuated by aberrant growth factor signaling pathway components, overexpression of cell cycle checkpoint proteins (especially CDK4), and aberrant activity of transcription factors including NF-κB (16–18). Patient-derived cell lines and a xenograft model showed sensitivity to blocking NF-κB through use of the proteasome inhibitor bortezomib (18, 19). CDK4/6 inhibitors such as abemaciclib and palbociclib have also shown promising preclinical activity (20) and are being evaluated in clinical trials (21). However, there are currently no clinically approved targeted therapies for chordoma. Selinexor and eltanexor are selective inhibitor of nuclear export (SINE) compounds that specifically inhibit the exportin-1 (XPO1, or CRM1) protein, a karyopherin that is often upregulated in human cancers (22–24). XPO1 mediates the nuclear export of multiple tumor suppressor proteins (TSPs) (25), and its inhibition causes TSP nuclear retention leading to decreased cancer cell proliferation and cancer cell apoptosis, while sparing healthy tissue (26–28). XPO1 also interacts with IκB, the endogenous inhibitor of NF-κB, and SINE-mediated inhibition of XPO1 suppresses NF-κB signaling. Selinexor has demonstrated anti-cancer activity in both solid and hematological malignancies (29), has been clinically approved for treatment of multiple myeloma (30) and diffuse large B-cell lymphoma (31), and is in advanced clinical trials for dedifferentiated liposarcoma and glioblastoma multiforme (32, 33). In this study, the XPO1 inhibitors, selinexor and eltanexor, were investigated as anti-cancer agents in five different chordoma patient-derived xenograft (PDX) models, as single agents or in combinations with bortezomib or abemaciclib.

## Materials and methods

### *In vivo* studies

*In vivo* studies were conducted through the Chordoma Foundation’s Drug Screening Program at XenoSTART (San Antonio, Texas) under International Animal Care and Use Committee (IACUC) approved protocols. Five chordoma PDX models were used: CF382 (recurrent clival chordoma, 57-year old female); CF466 (metastatic lumbar chordoma, 58-year old male); SF8894 (recurrent clival chordoma, 59-year old male); CF459 (primary clival chordoma, <20-year-old male); and CF365 (poorly differentiated metastatic clival chordoma, 11-year-old male). PDX fragments from host animals (~70mg) were implanted subcutaneously into the right flank of 6~12-week-old female NSG mice (NOD.CgPrkdc(scid)ll2rg(tm1Wjl)SzJ) purchased from The Jackson Laboratory or athymic nude mice (Crl : NU(NCr)-Foxn1nu) purchased from Charles River Laboratories. Tumor volume (TV) and animal weight data were collected twice a week electronically using a digital caliper and scale, respectively. Mice were housed under standard conditions (Teklad 2919 irradiated feed and water given ad libitum; 30-60% humidity; 21- 24°C; 12h light daily). All animal studies were carried out under protocols approved by the XenoSTART IACUC Committee. Once tumors reached a TV of at least 150-300 mm^3^, animals were matched by TV and randomized to control (untreated) and treatment groups (n=5 mice per group). Mice were dosed by oral gavage using an 18-gauge curved ball-bearing syringe tip with selinexor (5 mg/kg, 4 times weekly, PO), or eltanexor (10 mg/kg; 5 times weekly PO), as single agents or in combination with the proteasome inhibitor bortezomib (0.3 mg/kg, twice weekly, IV [tail vein injection]) or the CDK4/6 inhibitor abemaciclib (50 mg/kg, daily, PO) for 6 weeks. Significance for differences in tumor size was determined by one-way ANOVA. After 6 weeks, tumors were collected for gross and histological biomarker analyses as well as gene expression profiling.

### Compounds

Selinexor was obtained from Karyopharm Therapeutics. Bortezomib (#S1013) was purchased from Selleckchem. Abemaciclib was purchased by XenoSTART.

### Histological and immunohistochemistry analysis

Tumor samples from mouse PDX models were fixed in 10% neutral buffered formalin, processed and paraffin embedded. Four-micron sections were stained with hematoxylin & eosin (H&E, Richard-Allen Scientific) for routine histology. For immunohistochemistry (IHC), 4 µm sections were baked on slides at 65°C for 30 min, deparaffinized and rehydrated, placed in Declare working buffer, steam-cooked for antigen retrieval, cooled, and transferred to 3% hydrogen peroxide to block endogenous hydrogenase activity. Protein block was applied before primary antibodies were incubated with slides. Cell Marque Hi-Def Polymer Amplifier and secondary antibody were applied sequentially at room temperature as per manufacturer’s instructions. DAB chromogen was used for color reaction. Slides were counterstained with hematoxylin, dehydrated, mounted, and cover-slipped. IHC staining was performed on a Biogenex I6000 automated stainer. Digital images of the slides were obtained through an Aperio AT Turbo slide scanner at 20×. Primary antibodies against XPO1 (Bethyl Laboratories, A300-469A, 1:15k), Brachyury (Abcam, ab209665, 1:30k), APC (Abcam, ab15270, 1:3k), FOXO3A (Cell Signaling Technology, 12829, 1:1k), eIF4E (Protein Tech, 11149-1-AP, 1:500), Survivin (Abcam, ab76424, 1:1k), SOX9 (Sigma-Aldrich, HPA001758, 1:1k), YAP1 (Cell Signaling Technology, 14074, 1:500), PARP1 (Santa Cruz, sc-8007, 1:1k), Ki67 (Biocare, Prediluted), SMAD4 (Santa Cruz, sc-7966, 1:600), and cleaved Caspase 3 (Cell Signaling Technology, #9661, 1:1k) were used for IHC analysis. Cell number and IHC staining intensity were quantified with Aperio image analysis algorithms. H-Score was calculated for nuclear-stained biomarkers.

### Western blotting

Cells were seeded in 6-well plates at a density of 0.5×10^6^ cells/well and allowed to adhere overnight. Post-treatment, the cells were washed with PBS and then lysed with RIPA buffer (#89901, Thermo Scientific) supplemented with protease inhibitors (# 05892791001, Roche) and phosphatase inhibitors (#04906837001, Roche). The protein level of each sample was quantified and normalized using BCA assay (#23225, Thermo Scientific). 20 μg of each sample were run in 4-12% Bis-Tris Gel (Life Technologies) and later transferred to nitrocellulose membrane using iBlot Gel Transfer Kit (Life Technologies). The membranes were blocked using LI-COR blocking buffer (#927-40000, LI-COR), probed with the indicated antibodies (XPO1 [sc-5595], MDK1 [sc-46701] and β-actin [sc-81178], Santa Cruz Biotechnology; PLCD1 [#3832], Cell Signaling Technology) and analyzed using Licor Odyssey.

### Gene expression profiling and analysis

RNA extraction from formalin-fixed paraffin-embedded tumors and sequencing were performed by Novogene Corporation, Ltd. (Beijing, China) or Psomagen, Inc. (Rockville, MD). Reads were assessed for quality using FastQC (Babraham Bioinformatics, Cambridge, UK) then aligned to the human genome build 38 using HISAT2 (34). Raw gene-level counts were determined using FeatureCounts (35). Normalization and differential expression analysis was performed by fitting a negative binomial model using DeSeq2 (36). As a quality control, variance stabilizing transformation was applied to the norm counts, then principal components were visualized to confirm that no samples were technical outliers. Each drug treatment group was compared to the vehicle-treated group of the same PDX model to calculate: base mean expression, log_2_ fold change and corresponding standard error, Wald statistic, Wald test p-value and Benjamini-Hochberg adjusted p-value. Pathway analysis was performed using Ingenuity Pathway Analysis (IPA) software (Qiagen, Hilden, Germany) to compare selinexor-treated tumors to vehicle treated tumors by using the adjusted p-value and log_2_ fold changes from DeSeq2. Cut offs of P_adj_ < 0.05 and fold change > 0.5 or < -0.5 were applied for the analysis. Gene Set Enrichment Analysis was performed to compare the selinexor treated vs. vehicle treated tumors by using rank-ordered lists of the Wald statistics for all expressed genes. The MSigDB canonical pathways from Pathway Interaction Database were used as the reference pathway set (37).

### Statistical analysis

Tumor size comparisons were performed on the final day of measurements (day 42) using one-way ANOVAs and Sidak’s multiple comparisons tests, comparing all treatment groups. Differences in H-scores were determined using t-tests. For RNAseq data, gene-level Benjamini-Hochberg adjusted p-values are presented showing significance for differences between each treatment group and the corresponding control group. The top five pathways are presented from the IPA analysis, regardless of overall raw p-value enrichment of the pathway.

## Results

### SINE compounds and combinations reduced PDX tumor growth

**Figure 1:**
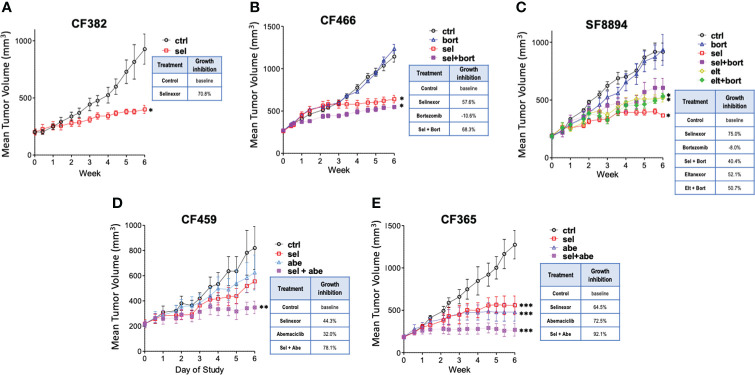
Selinexor inhibits chordoma growth in five patient derived xenograft models. Tumor volume over time determined in **(A)** CF382; **(B)** CF466; **(C)** SF8944; **(D)** CF459; and **(E)** CF365 PDX models under untreated control, selinexor (sel), or eltanexor (elt) treatment either as single agents or in combination with bortezomib (bort) or abemaciclib (abe). Dosing: selinexor, 5 mg/kg, 4 times weekly, PO; eltanexor, 10 mg/kg; 5 times weekly, PO; bortezomib, 0.3 mg/kg, twice weekly, IV; abemaciclib, 50 mg/kg, daily, PO. Data are shown as mean +/- SEM. Relative percent change to control at termination of study is shown for each condition in inset tables. Significance determined using ANOVA with Bonferroni post-test comparing each experimental group to control. *p < 0.05; **p < 0.01; ***p < 0.001.

Five PDX models of different chordoma subtypes were used to assess *in vivo* efficacy of SINE compounds, with molecular characterization of post-treatment tumors (**Supplemental Figure S1**). First, mice bearing CF382 recurrent clival xenografts were treated using selinexor alone. After six weeks, selinexor treated mice had tumors that were significantly smaller than control untreated mice, with an average growth reduction of 70%, and minimal weight loss associated with drug toxicity (**Figure 1A** and **Supplemental Figure S2A**). As selinexor and the proteasome inhibitor bortezomib both act on the NF-κB pathway, and bortezomib can inhibit chordoma cell line proliferation (18, 19), we explored the possibility of additive/synergistic effects of combining these treatments. The CF466 model of metastatic sacral chordoma was obtained for these combination experiments to assess effectiveness against a different type of chordoma. Mice treated with selinexor alone or in combination with bortezomib had significantly smaller tumors after 6 weeks than control untreated mice, whereas bortezomib alone caused no difference in tumor volume compared to controls (**Figure 1B**). We next evaluated a model of recurrent clival chordoma, SF8894, using bortezomib combinations with 2 different SINE compounds, selinexor and eltanexor, which, at the time of the preclinical experiments, had just entered clinical evaluation. Similar to CF466, the SINE compounds alone and in combination with bortezomib significantly reduced tumor volume compared to untreated mice. Interestingly, animals bearing SF8894 tumors treated with selinexor as a single agent showed greater reduction in tumor growth compared to those with the selinexor/bortezomib combination, although this was not statistically significant (**Figure 1C**). Although eltanexor significantly reduced tumor volume compared to controls in the SF8894 model, eltanexor-treated tumors were larger than selinexor-treated tumors, and thus eltanexor was not explored further. Neither selinexor, eltanexor, nor bortezomib treated animals had significantly lower body mass compared to controls in these models (**Supplemental Figures S2B, C**). As CDK4/6 inhibitors act synergistically with other targeted therapies against chordoma *in vitro* (38), the efficacy of SINE compound combinations with the CDK4/6 inhibitor, abemaciclib, was also assessed. Again, to allow a comprehensive evaluation of multiple types of chordoma, two additional models were used for these experiments the metastatic poorly differentiated pediatric clival chordoma model, CF365, and the primary pediatric clival chordoma model CF459. Mice that were treated with selinexor or abemaciclib as single agents had smaller volume tumors compared to vehicle, with no loss in total animal mass (**Figures 1D, E** and **Supplemental Figures S2D, E**). The selinexor and abemaciclib combination resulted in the greatest tumor growth inhibition, demonstrating an additive (or synergistic) effect between the compounds (**Figures 1D, E**).

### Selinexor treated tumors showed nuclear retention of tumor suppressors and reduction in oncoprotein levels

**Figure 2:**
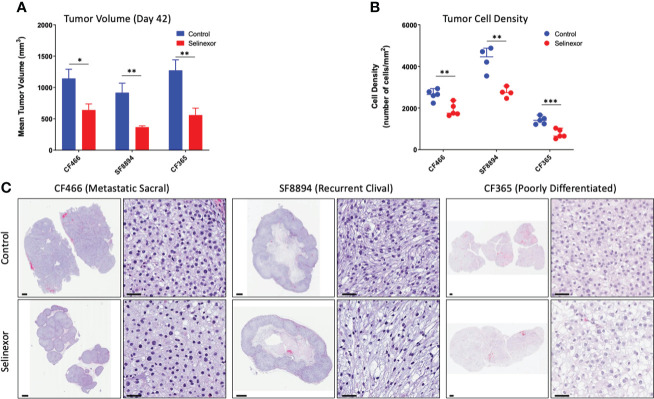
Selinexor treatment reduces both tumor size and tumor cell density in three different chordoma preclinical PDX models. **(A)** Tumor volume at termination of six week treatment; CF466 model, p=0.02, n=5; SF8894 model, p=0.01, n=4; CF365 model, p=0.007, n=5. Data shown as mean +/-SEM. **(B)** Tumor cell density at termination of six-week treatment, CF466 model, p=0.004, n=5; SF8894 model, p=0.003, n=4; CF365 model, p=0.0009, n=5. Data shown as individual data points, **(C)** H&E images of indicated tumor models and treatments at low and high magnifications with 1 mm and 50 um scale respectively. All P-values calculated using t-tests. *p < 0.05, **p < 0.01, ***p < 0.001.

**Figure 3:**
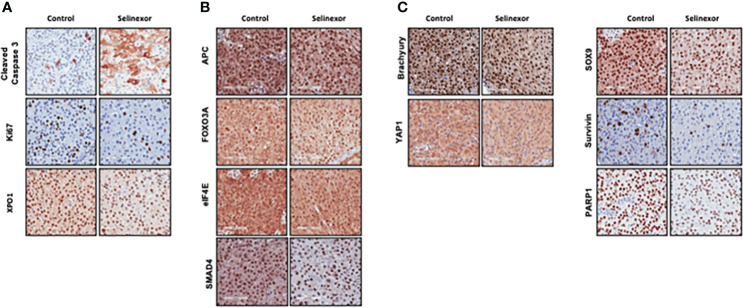
Effect of selinexor on indicators of cell health, chordoma markers, and XPO1 cargo proteins. Immunohistochemistry analysis of tumor samples from CF466 PDX models treated for six weeks with either vehicle control or selinexor. **(A)** Markers of cell survival (cleaved caspase 3) and proliferation (Ki67), as well as the selinexor target XPO1. **(B)** XPO1 cargo proteins APC, FOXO3A, eIF4E and SMAD4. **(C)** Proteins involved in sonic hedgehog signaling pathways such as SOX9, and YAP1, and regulators of chordoma cell growth, Brachyury and Survivin, as well as DNA repair enzyme PARP1.

IHC and histological examination was performed on excised tumors from several of these PDX models. Cell density was assessed in the CF466, SF8894, and CF365 models, and in all cases the tumors from selinexor treated mice had reduced density in addition to reduced tumor volume (**Figure 2**). In the CF466 PDX model, selinexor treated tissue shows increased expression of the apoptosis marker cleaved caspase 3 and decreased expression of cell proliferation marker Ki67 (n=3, p=0.088) (**Figure 3A**). As expected, XPO1 (n=4, p=0.15) protein expression was reduced and showed nuclear sequestration in treated samples (**Figure 3A**). The XPO1 cargo proteins SMAD4, APC, FOXO3A and eIF4E also showed an increase in nuclear localization, consistent with inhibition of XPO1 nuclear export activity (**Figure 3B**). SOX9 (n=4, p=0.04), whose knockdown was previously shown to inhibit chordoma cell growth and induce apoptosis (39), and PARP1 (n=5, p=0.004) and Survivin (n=3,p=0.047), also critical to chordoma cell growth (40), all show decreased expression with selinexor treatment (**Figure 3C**). Selinexor treatment may have led to a decrease in expression of Brachyury (n=4, p=0.037), a key driver of chordoma (41), as well as its downstream target YAP1 (**Figure 3C**). Quantification of IHC analysis is presented in **Supplemental Figure S3**.

### RNAseq revealed SINE compounds alter different pathways in different chordoma models

**Figure 4:**
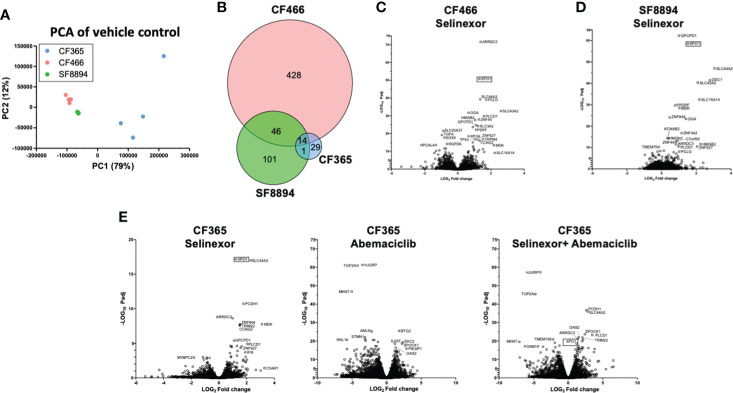
Differential expression analysis in treated xenografts compared to matched vehicle controls. **(A)** Principal component analysis (PCA) analysis of gene-level expressions in vehicle treated control tumors for the indicated PDX models. Each dot represents one tumor. **(B)** Venn diagram indicating the number of genes differentially expressed in selinexor treated tumors compared to match vehicle controls. **(C–E)** Volcano plots show comparison of all expression of genes between indicated treatment group and vehicle control for CF466 **(C)** SF8894 **(D)** and CF365 **(E)** models. Y-axis is significance and x-axis is fold change. Top significant genes are labeled. XPO1 is indicated with a box.

**Figure 5:**
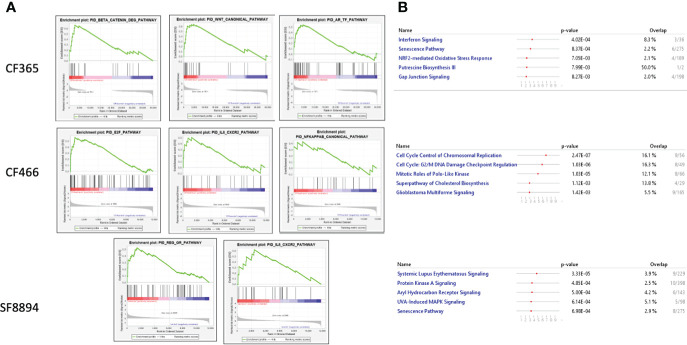
Top altered pathways in selinexor treated tumors. **(A)** Heatmaps of gene set enrichment analysis (GSEA) show altered disease and function pathways and sub-pathways in CF466, SF8894 and CF365 xenografts treated with selinexor. Size of individual boxes are inversely proportional to P-value. Color is according to z-score. Produced using Ingenuity Pathway Analysis software. **(B)** IPA analysis of top modified pathways; top panel, CF365; middle panel, CF466; bottom panel, SF8894.

In addition to the focused assessment of protein levels and subcellular localization, we performed total transcriptome RNA sequencing on excised tumors from the CF466, SF8894, and CF365 PDX models to identify differentially expressed genes between control untreated mice with those treated with either selinexor alone, abemaciclib alone, or the combination. Principal component analysis (PCA) of vehicle treated tumors showed the CF466 and SF8894 cells had similar transcriptional profiles and the CF365 (poorly differentiated subtype) clustered separately (**Figure 4A**). Selinexor as a single agent induced altered expression of a substantial number of genes, with 488, 162 and 44 differentially expressed genes in the CF466, SF8894 and CF365 models, respectively (multiple test correction adjusted P-value [P_adj_] <0.01, **Figure 4B**, **Supplementary Tables 1–3**). Comparison of genes differentially expressed in selinexor treated mice that were common across two or more models revealed some similarities (**Figures 4C–E**). *XPO1* was one of the strongest upregulated genes in selinexor treated tumors, consistent with a known feedback loop that results from successful inhibition of XPO1 protein shuttling. Additionally, selinexor-treated tumors showed an increase in levels of tumor suppressor genes *PLCD* and *ARRDC3*, many solute carriers, the growth factor *MDK*, and the cell cycle regulator CCNG2, among others (**Supplementary Tables 1–3**, **Figures 4C–E**). The increase in the levels of PLCD and MDK was corroborated using chordoma cell lines by western blotting (**Supplementary Figure S4**). In abemaciclib-treated CF365 PDX tumors, we found 1335 differentially expressed genes compared to untreated control; the most significant were downregulation of the DNA topoisomerase TOP2A, the centromeric chaperone HJURP and the cell proliferation marker MKI67 (**Figure 4D**, **Supplementary Table 3**). Notably, many genes differentially expressed in single agent selinexor or abemaciclib treated CF365 PDX models were also differentially expressed in the combination treatment (**Supplementary Table 3**), indicating the treatments likely did not interfere with each other’s mechanisms of action. Pathway analysis revealed that selinexor-treated CF365 tumors had upregulation of the β-catenin degradation pathway, modulated WNT signaling and androgen receptor signaling, which is in line with the known effects of XPO1 inhibition in cancer cells (42) (**Figure 5A**, top panels). The top pathways affected by selinexor treatment in the CF466 model were the E2F pathway, IL8/CXCR2 inflammatory cytokine pathway and canonical NF-κB signaling, which are also known targets of XPO1 (43–45) (**Figure 5A**, middle panels). Interestingly, selinexor treatment of SF8894 cells also induced changes in IL8/CXCR2 signaling in addition to the glucocorticoid receptor (GR) pathway, which is in line with previous reports that selinexor up-regulates GR expression (46) (**Figure 5A**, bottompanels). Similar assessments with Ingenuity Pathway Analysis (Qiagen) revealed that the top canonical pathway changes in selinexor treated CF365 cells were related to interferons and senescence, in CF466 cell cycling and in SF8894 auto-immunity (systemic lupus signaling) and protein kinase A signaling (**Figure 5B**).

## Discussion

Our work demonstrated the effectiveness of the SINE compounds selinexor and eltanexor in reducing growth of PDX models of chordoma, when used as single agents or in combination with abemaciclib or bortezomib and identified specific RNA- and protein-level changes that occur in treated cells. These data highlight that selinexor could be an effective overall anti-cancer agent for treatment of chordoma patients, despite substantial underlying molecular heterogeneity between chordoma subtypes. Due to this heterogeneity, it is likely that the anti-cancer effects of selinexor are achieved through regulation of multiple pathways. IHC analysis of the metastatic sacral chordoma model CF466 showed increased nuclear retention of the XPO1 cargo SMAD4, which is an upstream regulator of Brachyury (47), and slightly reduced expression of both Brachyury and its downstream target YAP1, a hippo signaling pathway member that can control tumor stemness and aggressiveness (48). Expression of the Brachyury gene *TBXT* was also reduced by selinexor at the RNA level in the CF365 cells (**Supplementary Table 1**). Selinexor treatment led to increased nuclear retention and upregulation of the tumor suppressor proteins eIF4E and FOXO3A, and downregulation of an oncogene in the SHH signaling pathwaySOX9. Survivin and the DNA repair enzyme PARP1 were also downregulated after selinexor treatment in CF466 PDX models. Thus, SINE compounds likely exhibit multiple anti-cancer mechanisms in chordoma, consistent with their compound mechanisms of action in other malignancies. Compared with previous murine studies of selinexor’s anti-cancer activity, the experiments here used lower doses of selinexor administered more frequently, with animals dosed at 5 mg/kg four days per week, compared to previous studies using doses of 10 mg/kg two-three times weekly (49–52), 12.5mg/kg twice weekly (53), or 15 mg/kg two-three times weekly (54–56). This dosing schedule was effective at reducing tumor volumes when used alone or in combination with abemaciclib or bortezomib, and importantly, did not result in animal weight loss, a marker of adverse toxicity. Though eltanexor was able to significantly inhibit the growth of the SF8894 tumor model as a single agent, selinexor showed better efficacy than eltanexor. As a result, we moved forward with assessment of selinexor in additional models of chordoma. SINE compounds have synergistic effects in combination with bortezomib in multiple cancer types, yet the lack of synergy observed in the SF8894 and CF466 models was unexpected. Seeking to explore an additional combination agent, abemaciclib was selected as it is an approved CDK4/6 inhibitor, and chordomas have a near universal loss of CDKN2A and p16 resulting in activation of CDK4/6. We were able to obtain two additional PDX models for assessing the efficacy of this combination, as described. A strength of SINE compounds is simultaneous attenuation of multiple oncogenic pathways and overcoming cancer heterogeneity to a certain degree. The fact that selinexor can inhibit tumor growth of multiple subtypes of chordoma aligns with the specific mechanisms of action of SINE compounds. Taken together, a diverse spectrum of chordoma models played an important part in dissecting the anti-tumor activity of these compounds. RNA sequencing revealed differences in the baseline untreated transcriptional profiles of the three cell lines that were sequenced. The CF365 cell line, which was derived from a poorly differentiated chordoma characterized by loss of the BAF complex gene *SMARCB1*, clustered separately from the CF466 and SF8894 cells, both of which express SMARCB1 (57–59). Selinexor treatment of these tumors revealed both similarities and differences between the models. The tumor suppressor gene *ARRDC3* was markedly upregulated in all three models after selinexor treatment (**Figure 4**, **Supplementary Table 1** consistent with previous studies performed in triple negative breast cancer cells (60). Likewise, treatment resulted in upregulation of several solute carriers including the choline transporter SLC44A2, which was observed in prior investigations of selinexor effects on the transcriptome (61, 62). However, it remains unclear if this is a direct or indirect effect of XPO1 inhibition, and the role solute carries, or their substrates may have in selinexor-mediated anti-tumor effectiveness. Examining the levels of *XPO1* RNA in vehicle-control tumors showed that the CF365 model had lower expression compared to the CF466 and SF8894 models. However, selinexor had similar effectiveness in all models, indicating the effectiveness of the drug is not dependent on baseline RNA levels of *XPO1.* Notably, biomarker studies of selinexor have not shown a direct relationship between efficacy and baseline XPO1 RNA expression in any investigated tumor type. Interestingly, discrepancies between RNA and protein expression may be attributed to a cellular feedback loop that senses the inhibition of nuclear export activity and induces *XPO1* mRNA expression. However, this increased expression of *XPO1* mRNA does not translate into additional XPO1 protein. This explains how selinexor treatment reduced the level of XPO1 protein (**Supplementary Figure S4**) and at the same time increased *XPO1* mRNA (**Figure 4**). In fact, *XPO1* mRNA induction is commonly used as the pharmacodynamic marker for selinexor-mediated XPO1 inhibition in humans. Despite the similarities of some transcriptional changes between the three cell lines on which expression profiling was performed, the most significant pathway differences between control and selinexor treated mice were different for each of the three PDX models, with CF365 cells showing changes in WNT signaling, CF466 in E2F signaling, and SF8894 in GR signaling. Each of these pathways can drive oncogenesis when dysregulated, and interestingly, E2F and GR signaling have been shown to be targetable by XPO1 inhibitors (43, 46, 63). Specific to the CF466 sacral chordoma model, *TGFA* was among the most significantly reduced genes, which is notable because it encodes an EGFR ligand, and SINE compounds are effective against cancer cells with engineered resistance to EGFR-tyrosine kinase inhibitors (64). Notably, direct EGFR inhibitors have shown promise as anti-cancer agents in chordoma and are being clinically evaluated (65). Our findings demonstrate clinical stage XPO1 inhibitors may be effective agents for treatment of effectiveness when combined with the CDK4/6 inhibitor abemaciclib. Clinical investigation of a selinexor and abemaciclib combination for treatment of patients with chordoma is warranted.

## Data availability statement

The original contributions presented in the study are included in the article/**Supplementary Material**. Further inquiries can be directed to the corresponding author.

## Ethics statement

The animal study was reviewed and approved by XenoSTART IACUC Committee.

## Author contributions

TK, JL, and YL designed the study. MJW, HC, LH, TK, and JL oversaw animal experiments. CW and HC analyzed genomic data, and performed statistical testing. HC performed immunohistochemistry. SS and YL supervised the project. JL provided chordoma samples. All authors contributed to the article and approved the submitted version.

## Funding

JetPub Scientific Communications, LLC supported by funding from Karyopharm, provided drafts and editorial assistance to the authors during preparation of this manuscript.

## Acknowledgments

**Supplementary Figure S1** was created with BioRender.com.

## Conflict of interest

Authors CW, HC, LH, TK, SS, and YL are all current or former employees of Karyopharm. MJW was employed by XenoSTART.

The remaining authors declare that the research was conducted in the absence of any commercial or financial relationships that could be construed as a potential conflict of interest.

The authors declare that this study received funding from Karyopharm and the Chordoma Foundation. The funders had the following role in this study: data generation, analysis, and interpretation, writing of the manuscript and submitting it for publication.

## Publisher’s note

All claims expressed in this article are solely those of the authors and do not necessarily represent those of their affiliated organizations, or those of the publisher, the editors and the reviewers. Any product that may be evaluated in this article, or claim that may be made by its manufacturer, is not guaranteed or endorsed by the publisher.
