# Supplementary material for: Selinexor inhibits growth of patient derived chordomas in vivo as a single agent and in combination with abemaciclib through diverse mechanisms
Source: Front Oncol. 2022 Aug 18;12:808021. doi: 10.3389/fonc.2022.808021 (PMC9434827; doi:10.3389/fonc.2022.808021)
Supplement: Supplementary file 2 [file DataSheet_2.docx]

Supplementary Material

# Supplementary Figures

**Supplementary Figure S1**


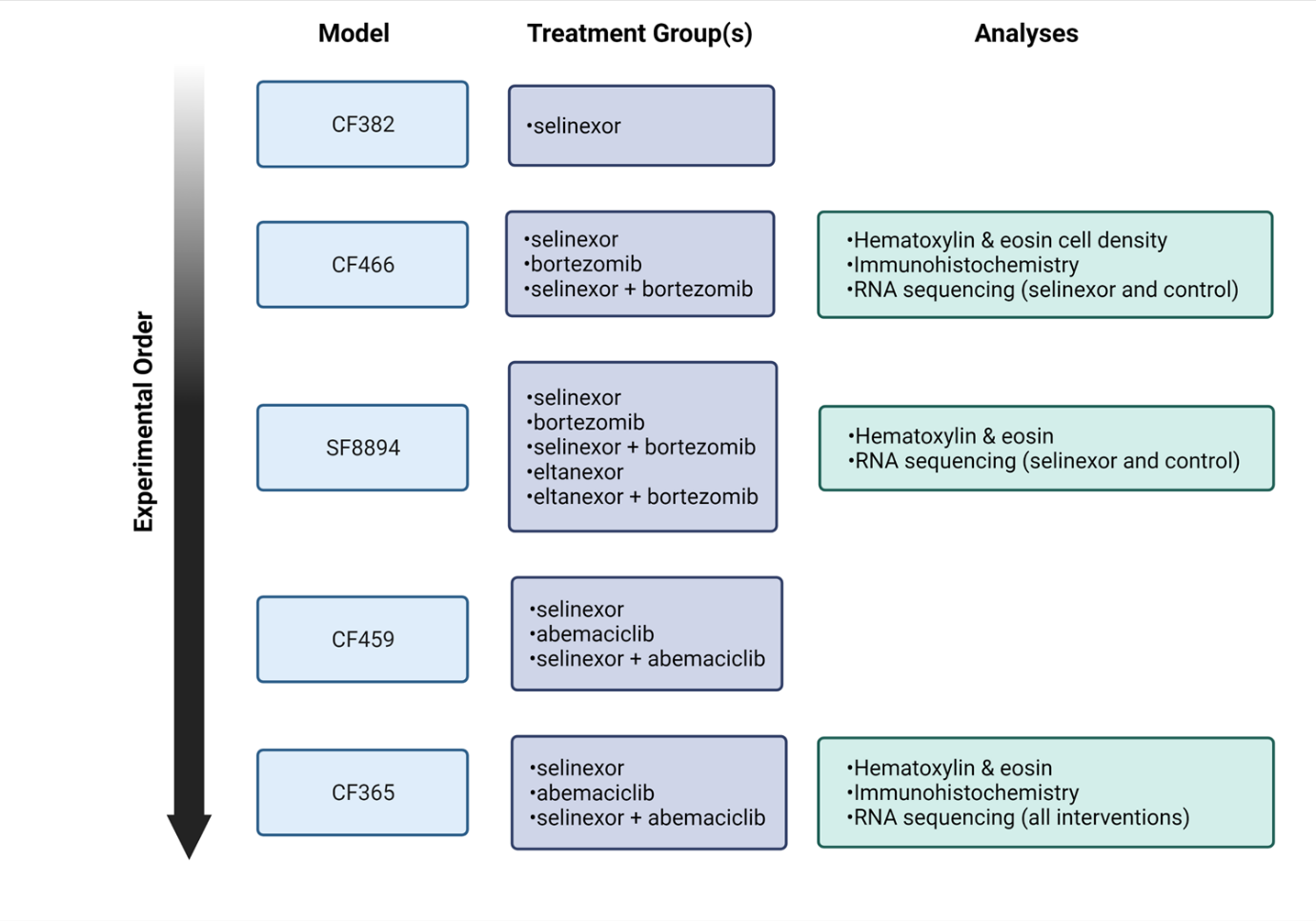


**Supplementary Figure S1.** **Experimental flowchart chordoma PDX models, treatments, and analyses**. CF382 (recurrent clival chordoma, 57-year old female); CF466 (metastatic lumbar chordoma, 58-year old male); SF8894 (recurrent clival chordoma, 59-year old male); CF459 (primary clival chordoma, <20-year old male); and CF365 (poorly differentiated metastatic clival chordoma, <20 year old male) with their corresponding analyses in experimental order.

**Supplementary Figure S2**


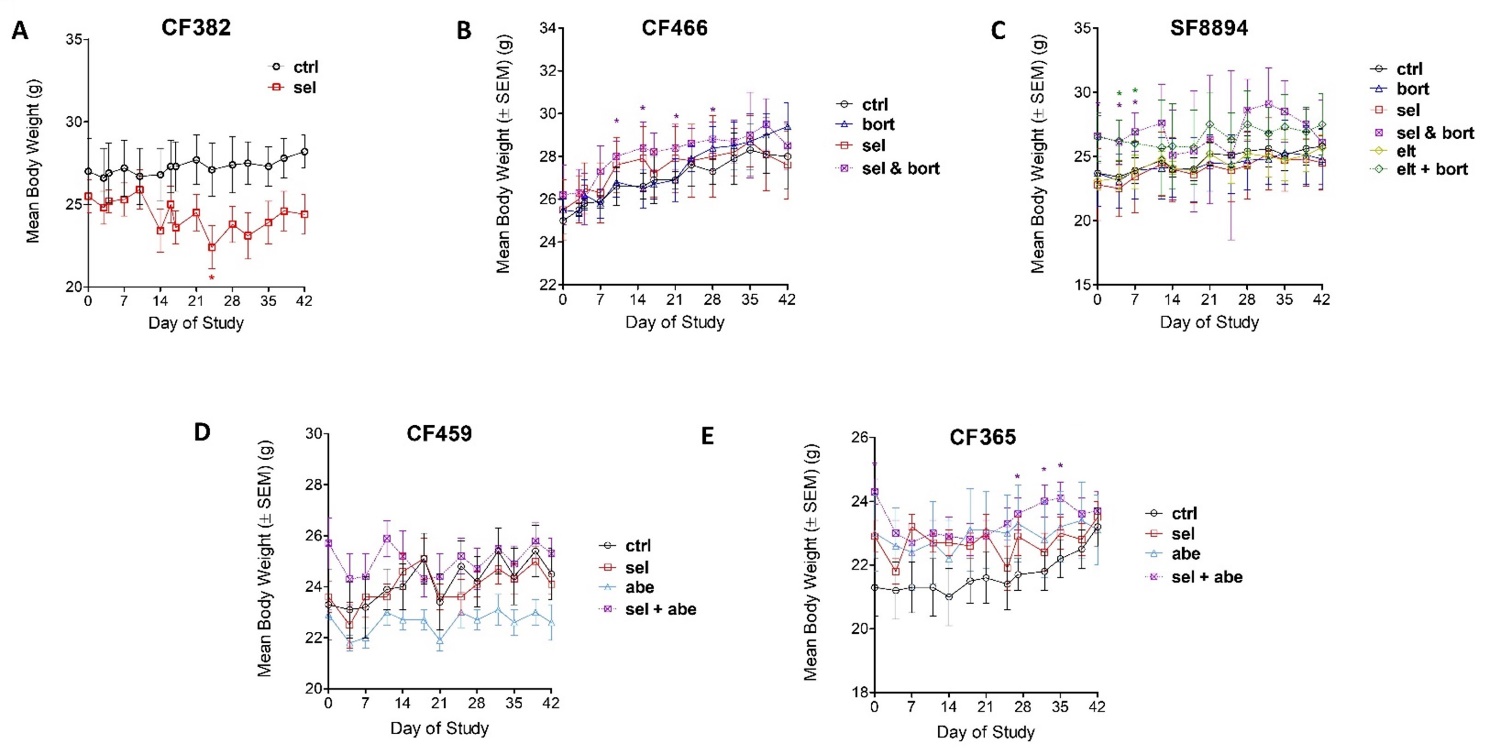


**Supplementary Figure S2: Mass of Treated Animals During Xenograft Studies.** Animal mass over time determined in **A,** CF382; **B**, CF466; **C**, SF8894; **D**, CF459; and **E,** CF365 PDX models under control or selinexor (sel) treatment either as single agent or in combination with bortezomib (bort) or abemaciclib (abe). Animals were dosed with selinexor as a single agent (5mg/kg, 4 times weekly, PO) or in combination with the proteasome inhibitor bortezomib (0.3mg/kg, twice weekly, IV) or the CDK4/6 inhibitor abemaciclib (50mg/kg, daily, PO). Data are shown as mean ± SEM. Significance tested at each time point by comparing each treatment group to vehicle-treated control using t-tests. Colors of asterisks correspond to groups. *p<0.05, **p<0.01, ***p<0.001.

**Supplementary Figure S3**


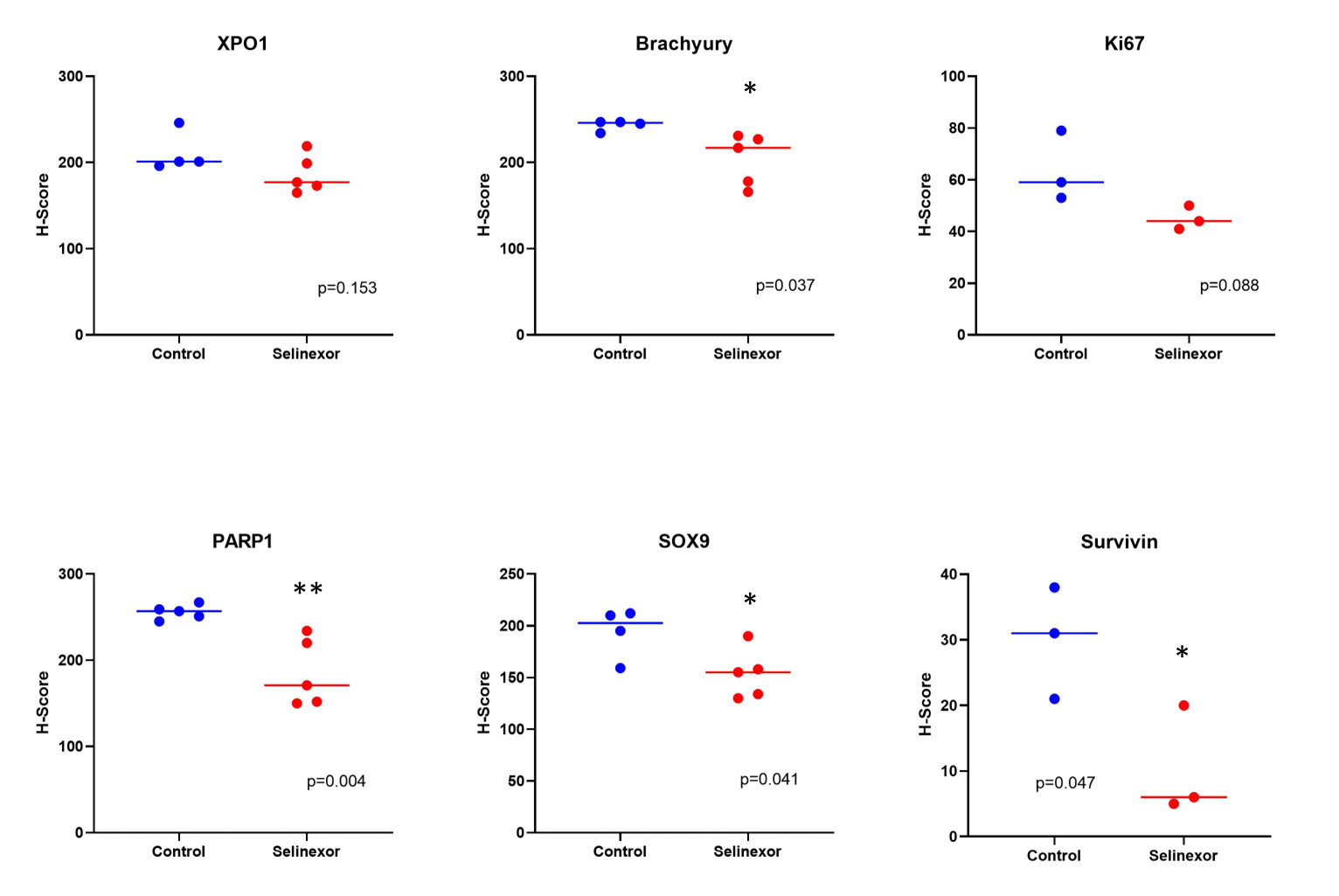


**Supplementary Figure S3: Quantification of IHC staining for relevant nuclear biomarkers presented in Figure 3.** H-score was calculated using Aperio image analysis software, using the equation: H-score=(3+)%*3+(2+)%*2+(1+)%; cells were categorized as 3+ (strong), 2+ (moderate), 1+ (weak), and 0 (negative) based on IHC staining intensity. H-scores were determined for representative images from 3-5 tumors/group, and compared between control and selinexor treated xenografts. Significance determined by t-test. *p <0.05, **p<0.01

**Supplementary Figure S4**


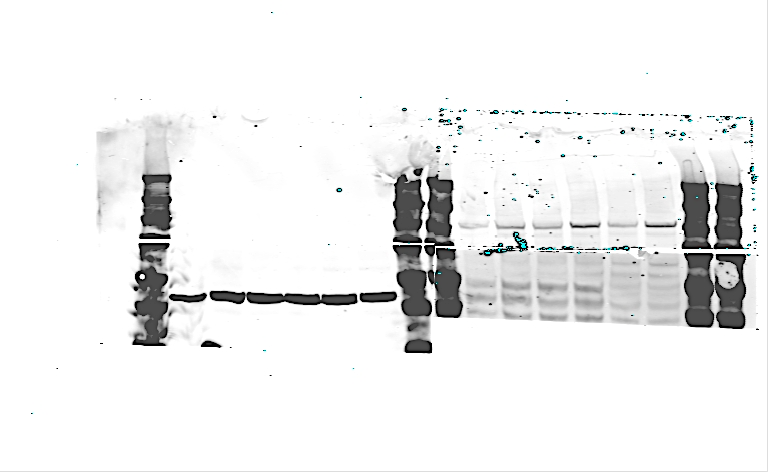

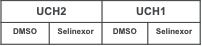


**XPO1**


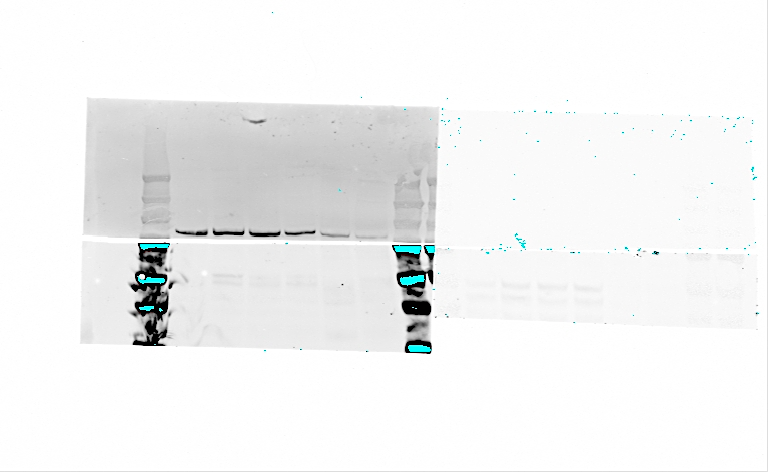


**PLCD1**

**MDK**


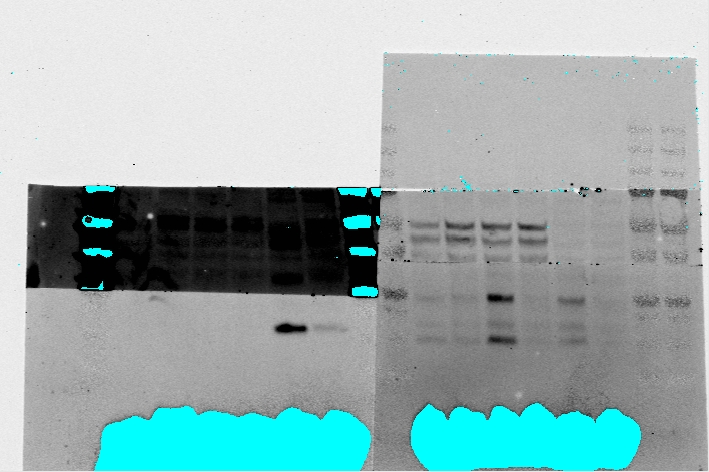


**Beta-Actin**


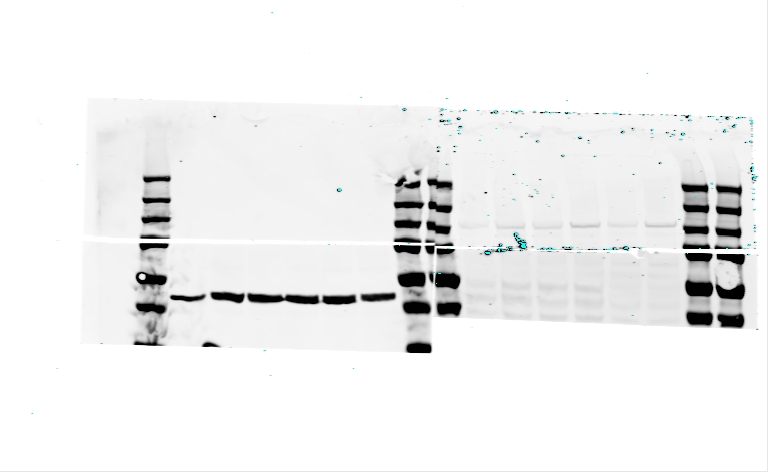


**Supplementary Figure S4: Selinexor induces the protein levels of PLCD1 and MDK in Chordoma cell lines.** Chordoma cell lines, UCH1 and UCH2, were treated with selinexor at a concentration equivalent to the IC_50_ for 24 hours. Protein levels of XPO1, PLCD1, and MDK were analyzed by western blotting. Selinexor treatment led to reduction in the levels of XPO1 and induction in the levels of PLCD1 and MDK. **Supplementary Tables**

**Supplementary Table 1. Significantly differentially expressed genes in CF466 model (selinexor vs vehicle control)**

- (See additional Excel file)

**Supplementary Table 2. Significantly differentially expressed genes in SF8894 model (selinexor vs vehicle control)**

- (See additional Excel file)

**Supplementary Table 3. Significantly differentially expressed genes in CF365 model**

- (See additional Excel file)
